# Sleep Deprivation Diminishes Attentional Control Effectiveness and Impairs Flexible Adaptation to Changing Conditions

**DOI:** 10.1038/s41598-017-16165-z

**Published:** 2017-11-22

**Authors:** Paul Whitney, John M. Hinson, Brieann C. Satterfield, Devon A. Grant, Kimberly A. Honn, Hans P. A. Van Dongen

**Affiliations:** 1 Department of Psychology, Washington State University, Pullman, WA 99164-4820 USA; 2 Sleep and Performance Research Center and Elson S. Floyd College of Medicine, Washington State University, Spokane, WA 99210-1495 USA; 3 Department of Psychiatry, College of Medicine, University of Arizona, Tucson, AZ 85721 USA

**Keywords:** Cognitive neuroscience, Predictive markers

## Abstract

Insufficient sleep is a global public health problem resulting in catastrophic accidents, increased mortality, and hundreds of billions of dollars in lost productivity. Yet the effect of sleep deprivation (SD) on decision making and performance is often underestimated by fatigued individuals and is only beginning to be understood by scientists. The deleterious impact of SD is frequently attributed to lapses in vigilant attention, but this account fails to explain many SD-related problems, such as loss of situational awareness and perseveration. Using a laboratory study protocol, we show that SD individuals can maintain information in the focus of attention and anticipate likely correct responses, but their use of such a top-down attentional strategy is less effective at preventing errors caused by competing responses. Moreover, when the task environment requires flexibility, performance under SD suffers dramatically. The impairment in flexible shifting of attentional control we observed is distinct from lapses in vigilant attention, as corroborated by the specificity of the influence of a genetic biomarker, the dopaminergic polymorphism DRD2 C957T. Reduced effectiveness of top-down attentional control under SD, especially when conditions require flexibility, helps to explain maladaptive performance that is not readily explained by lapses in vigilant attention.

## Introduction

Sleep loss is a frequent experience in modern life, but people tend to underestimate its impact^1–5^. Controlled studies show that sleep deprivation (SD) can have profound, adverse effects on cognitive functioning. Cognitive impairment during SD results from increasing sleep drive across time awake (sleep/wake homeostasis), modulated by time of day (circadian rhythmicity)^6–8^. Individuals differ systematically in their vulnerability to performance impairment during SD^9,10^, and genes have been identified that are associated with this differential vulnerability to sleep loss^11–13^.

However, the degree of impairment from SD varies widely not only across individuals but also among cognitive tasks^14,15^. The effects of SD are particularly potent for tests of vigilant attention, while tests of working memory, decision making, and executive functioning show smaller, more inconsistent effects^2,16,17^. It has been argued that deficits in vigilant attention are a universal root cause of cognitive impairments associated with sleep loss^2^. Yet, deficits in vigilant attention alone do not account for the wide range of problems in cognition and performance that accompany sleep loss^3,18^. Important consequences of SD observed in critical real-world settings such as disaster management, hospital care, and military operations – including poor decision making, loss of situational awareness, and perseverative behavior – await a coherent scientific explanation. In such real-world settings, where sleep loss can have its most dramatic impact, performance generally depends on management of multiple demands on information processing. Several studies have tested whether such performance issues produced by SD may result from declines in working memory (WM) capacity^19,20^. While overall performance on tests of WM typically declines under SD, the effects do not appear to be specific to WM capacity. For example, SD effects in WM tasks do not increase incrementally with increases in load, such as when items in a list must be retained three items back versus one item back^14^. Several investigators have suggested that instead of lowering WM capacity, SD may impair top-down attentional control^21–23^.

Top-down control is used when the predictable structure of the environment allows for anticipation of upcoming events or the outcomes of choices based on expectations. In turn, when conditions change and expectations are violated, top-down control can aid in the detection of change and updating of goals^24^. Effective attentional control requires a balance between taking advantage of stable and predictable features of the environment, and flexible reallocation of attention to fit changing environmental conditions.

We previously reported that susceptibility of decision making to impairment from SD depends on whether decisions require flexible reallocation of attention^25^. Specifically, subjects performing a task while deprived of sleep were unable to adapt to a reversal of stimulus–response mappings. This failure to adapt resulted in perseverative responding and large numbers of errors. The finding implicates flexible attentional control as a source of impairment resulting from SD.

**Figure 1:**
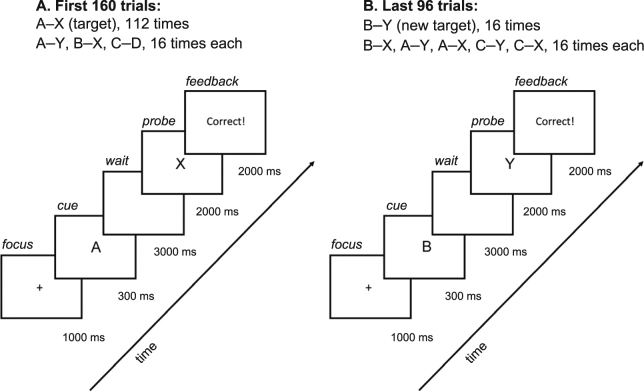
Schematic of the AX-CPT-s. (**A**) The first 160 trials (pre-switch) represented a standard implementation of the AX-CPT. Subjects were to respond with a left mouse click to an “X” probe whenever it followed an “A” cue, and a right mouse click for all other cue–probe combinations. The target “X” probe followed the “A” cue on 70% of the trials. (**B**) In the version of the AX-CPT used here, the standard trials were followed by 96 additional trials (post-switch), for which subjects were informed that they were to switch the response pattern—so that they now were to respond with a left mouse click to a “Y” probe whenever it followed an “B” cue, and a right mouse click for all other cue–probe combinations. The other cue–probe combinations included foils involving the old cue (“A”) and/or probe (“X”), with all different combinations presented equally often. The foils allowed investigation of interference effects from the previously valid cue and probe.

The present study was designed to allow us, within a single task platform, to examine the effects of SD on both the ability to effectively use top-down control *and* the ability to flexibly shift attentional control under changing conditions. Top-down control of attention has been studied in a variety of populations and circumstances using the AX Continuous Performance Task (AX-CPT; Fig. 1A)^26–29^, which requires subjects to make a target response (e.g. left keypress) for the valid cue-probe combination (A–X) and a different, non-target response (e.g. right keypress) for all other cue-probe combinations (e.g. B–X). In a typical implementation of the AX-CPT, the A cue is followed by the X probe on 70% of trials. Under such conditions, healthy, rested, young adults show a consistent pattern of using top-down attentional control (also known as proactive control) on the AX-CPT by anticipating that an X will occur after an A cue and preparing the A-X response. This results in a high hit rate on A-X trials and a low false alarm rate on B-X trials because the invalid cue does not result in pre-activation of the A-X response. However, this top-down control strategy produces a disadvantage on A-Y trials, which leads to false alarms as the response for A-X is anticipated when the A-cue is presented. We also included trials with both invalid cues and invalid probes (C-D trials) to be able to detect any performance deficits not specifically related to top-down attentional control.

By evaluating responses to A-X, B-X, A-Y and C-D trials, the typical implementation of the AX-CPT allowed us to determine how SD affects the use of top-down control under standard conditions in which the contingencies remain stable over trials. Furthermore, we added a novel component to the task to determine whether attentional control strategies could shift flexibly when required to adapt to a change in cue-probe mappings. In our novel task version, the AX-CPT-s, we added trials in which the cue–probe contingency was switched, i.e. a switch from A–X to B–Y as the valid cue–probe set (Fig. 1B). The trial block after the contingency switch included distractor trials combining the new cue–probe set with elements from the old cue–probe set. Thus, using the same task platform, we assessed whether SD affects the use of top-down control (pre-switch), and whether attentional control could shift flexibly when cue-probe response contingencies are changed (post-switch). The ability to target specific cognitive control operations, and dynamic adjustments to these operations when conditions change, was a primary focus of the present study of the effects of SD on cognitive functioning.

Signal detection statistics based on the discriminability index d’^29^ were calculated on the AX-CPT-s data for pre-switch and post-switch trials (Table 1). For pre-switch trials, we calculated commonly reported d’ values using hit rates to the target cue–probe combination and false alarm rates to specific non-target cue–probe combinations^26^. Effective engagement of top-down attentional control will produce higher performance on the *X-probe d’ index* and lower performance on the *A-cue d’ index*. Because SD subjects could be expected to show deficits in vigilant attention unrelated to top-down control, we also calculated a *vigilant attention d’ index*. For the novel post-switch trials, we developed several indices following similar logic to the pre-switch trials. The *flexibility d’ index* measures the overall ability to distinguish the new cue–probe combination from the old one. Reductions on the other two indices, *new cue d’ and new probe d’*, isolate potential contributors to flexibility problems – interference from the old target when the valid new cue is presented, and interference from the old cue when the valid new probe is presented, respectively.

**Table 1:** Signal Detection Indices Diagnostic of Changes in Attentional Control on the AX-CPT-s and the Hits and False Alarm Data from Which They are Derived (Session 2).*

Index	Hits	FAs	*Sleep Deprivation*		*Control*	
			%Hits	%FAs	%Hits	%FAs
***Diagnostic cue-probe combinations for pre-switch (standard) trials***						
*A-cue d’*	A–X	A–Y	93.1 ± 6.2	40.4 ± 15.3	96.2 ± 3.4	41.2 ± 16.6
*X-probe d’*	A–X	B–X	93.1 ± 6.2	16.6 ± 12.5	96.2 ± 3.4	2.3 ± 2.3
*vigilant attention d’*	A–X	C–D	93.1 ± 6.2	7.7 ± 7.7	96.2 ± 3.4	1.7 ± 1.8
***Diagnostic cue-probe combinations for post-switch (cue–target shift) trials***						
*flexibility d’*	B–Y	A–X	85.0 ± 13.0	10.9 ± 12.5	94.8 ± 7.6	1.3 ± 1.3
*new cue d’*	B–Y	B–X	85.0 ± 13.0	23.1 ± 20.2	94.8 ± 7.6	19.7 ± 15.5
*new probe d’*	B–Y	A–Y	85.0 ± 13.0	15.8 ± 12.9	94.8 ± 7.6	3.7 ± 6.3

*FAs: False Alarms. %Hits: percentage of hits (mean ± SD). %FAs: percentage of false alarms (mean ± SD).

Forty-nine healthy adults (aged 27.3 ± 4.8 years; 22 women, 27 men) participated in a laboratory study. Subjects were randomized to a SD group (n = 34) or a control group (n = 15). The AX-CPT-s was administered at baseline (session 1) after a 10-hour sleep opportunity, and again 24 hours later (session 2, same time of day) after 31.5 hours of continuous wakefulness (SD group) or while well-rested after another 10-hour sleep opportunity (control group).

## Results

### Baseline AX-CPT-s data pre- and post-switch

**Figure 2:**
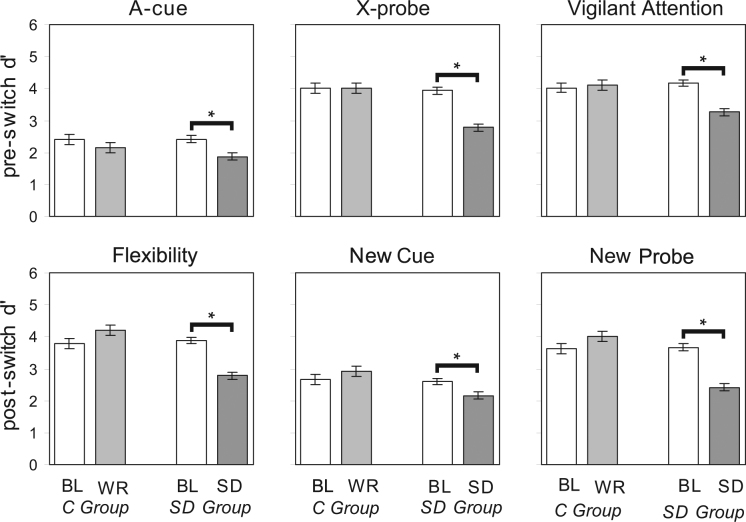
Performance on the AX-CPT-s in the control and SD groups. Panels show d’ for each of the performance indices described in Table 1, in session 1 (BL, baseline) and session 2 (WR, well-rested) in the control (*C*) group; and in session 1 (BL) and session 2 (SD, sleep-deprived) in the sleep deprivation (*SD*) group. Error bars are ± 1 standard error of the mean (SEM). Brackets indicate statistically significant contrast comparing session 1 to 2 (*p* < 0.05).

In session 1 (well-rested baseline) for both the SD group and the control group, the pre-switch data replicated the pattern typically observed with healthy young adults: predominant use of proactive control, with high *X-probe d’* and relatively low *A-cue d’* ^29^. Furthermore, the *vigilant attention d’* was high, indicating task performance did not suffer from attentional lapses. The post-switch data in session 1 showed continued use of proactive control while quickly adapting to the new contingencies, as indicated by high *flexibility d’* and relatively low *new cue d’*, while the *new probe d’* was high (Fig. 2). Thus, at baseline subjects could flexibly adapt attentional control in changing circumstances.

### Pre- and post-switch AX-CPT-s data after sleep deprivation or well-rested control

In session 2 (SD or well-rested control), there were substantial changes in the d’ indices relative to session 1 in the SD group as compared to the control group. Multivariate analysis of variance (MANOVA) of d’ index (6 levels) by group (2 levels) by session (2 levels) showed significant main effects of d’ index (*F*_5,48_ = 108.7, *p* < 0.001), group (*F*_1,48_ = 7.6, *p* = 0.008), and session (*F*_1,48_ = 12.2, *p* = 0.001). The analysis also showed a significant group by session interaction (*F*_5,48_ = 23.9, *p* < 0.001), demonstrating that SD degraded performance on the AX-CPT-s. Importantly, the index by group by session interaction was also statistically significant (*F*_5,48_ = 4.4, *p* = 0.002), indicating that the effects of SD on the d’ indices were not uniform (Fig. 2).

The effects were therefore investigated further for each d’ index separately. The interaction between group and session was statistically significant in all cases except for the *A-cue d’ index*, and for the *new cue d’ index* there was a trend. The statistical results are provided in Table S1 and described further below. The raw data (hits and false alarms) for session 2 from which the d’ indices were derived are in Table 1. Statistical results in the two sections below reflect head-to-head comparisons between session 2 (SD) and session 1 (baseline) in the SD group based on planned contrasts. For subjects in the control group, session 2 performance was not significantly different from session 1 performance based on planned contrasts (*F*_1,47_ ≤ 2.4, *p* ≥ 0.13).

### Sleep deprivation effects on pre-switch effectiveness of top-down attentional control

For subjects in the SD group, session 2 pre-switch data still showed the general pattern indicative of top-down attentional control – higher X-probe than A-cue performance – but both indices showed a significant drop from baseline performance (*A-cue d’*: *F*_1,47_ = 20.8, *p* < 0.001; *X-probe d*’: *F*_1,47_ = 58.4, *p* < 0.001). This pattern indicates that in the SD subjects, the use of top-down control was less effective in session 2 than in session 1. That is, despite showing a pattern consistent with top-down control, the subjects were less effective in preventing interference from a non-target probe compared to the rested conditions. In addition, there was evidence that lapses of attention (*vigilant attention d’*) reduced overall task performance (*F*_1,47_ = 43.2, *p* < 0.001) (Fig. 2).

### Sleep deprivation effects on post-switch flexibility of top-down attentional control

For subjects in the SD group, session 2 post-switch data showed an overall decline in the *flexibility d’* index (*F*_1,47_ = 32.0, *p* < 0.001) compared to baseline, though the absolute level of the *flexibility d’* performance was consistent with reasonably good ability to switch to the new valid cue-probe combination (B-Y). In addition, performance decreased on *any* trial that included either an old cue, reflected in the *new cue d’ index* (*F*_1,47_ = 4.5, *p* = 0.039), or an old target probe, reflected in the *new probe d’ index* (*F*_1,47_ = 31.8, *p* < 0.001). Thus, subjects in the SD group updated task-relevant information when it changed post-switch, but they could not effectively filter out the old, now task-irrelevant information. Anything that carried over from pre-switch trials caused interference, resulting in significant declines in d’ for all post-switch performance indices (Fig. 2).

### Converging evidence: Sleep deprivation effects on the Attention Network Test

Additional evidence that subjects were more susceptible to interference from a competing response during SD was obtained from the Attention Network Test (ANT)^30,31^. The ANT provided measures of the ability to use an alerting cue to detect a stimulus (alerting effect), use a cue to shift the location of visual attention (orienting effect), and manage response conflict (conflict effect). For subjects in the control group, performance did not significantly change across sessions (*F*_1,93_ ≤ 0.3, *p* ≥ 0.60) (Fig. S1). Under SD, however, we observed slower orienting (*F*_1,93_ = 5.3, *p* = 0.024), which may be related to attentional lapsing^32^. Importantly, under SD we found increased response conflict (*F*_1,93_ = 16.6, *p* < 0.001) (Fig. S1). Although the ANT was administered 5 hours earlier in the day than the AX-CPT-s in both test sessions, and the magnitudes of performance effects are therefore not directly comparable between the two tasks, the effect of SD on response conflict on the ANT is consistent with the effect we observed for attentional flexibility on the AX-CPT-s.

### Genetic stratification of sleep deprivation effects on flexibility in top-down attentional control

To investigate our finding that the effects of SD on the different d’ indices of the AX-CPT-s were not uniform (Fig. 2; see MANOVA results above), we investigated whether the effects varied differentially by a common genetic single nucleotide polymorphism (SNP) of the dopamine D2 receptor, DRD2 C957T (rs6277). This polymorphism has a strong effect on striatal dopamine receptor D2 binding potential^33^. Previous studies have associated it with variations in cognitive flexibility^34,35^, suggesting that any differences in susceptibility to SD related to this gene should be specific to the flexibility indices of the AX-CPT-s. Any such associations of SD performance with DRD2 that are specific to the flexibility indices would help establish that these effects on not simply downstream consequences of problems with vigilant attention. To determine differences in the SD effects associated with the DRD2 C957T genotype, we subdivided our SD sample into three allele groups (C/C, C/T, and T/T) for further analysis. The large SD effect sizes we observed (Fig. 2) permitted a well-powered test of gene–performance relationships.

**Figure 3:**
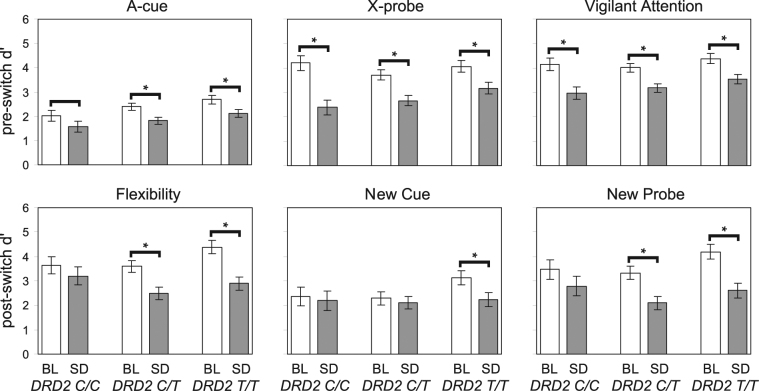
Performance on the AX-CPT-s in the SD group by genotype. Panels show d’ for each of the performance indices described in Table 1, in session 1 (BL, baseline) and session 2 (SD, sleep-deprived) for subjects homozygous for the C allele (*C/C*), heterozygous (*C/T*), or homozygous for the T allele (*T/T*) of the DRD2 C957T polymorphism. See Table 1 for interpretation of d’ changes. Error bars are ± 1 SEM. Brackets indicate statistically significant contrast comparing session 1 to 2 (*p* < 0.05); bracket without asterisk approaches significance (*p* = 0.10).

The effect of SD on post-switch performance was substantially influenced by genotype (Fig. 3). Relative to baseline, subjects with the T/T genotype were particularly vulnerable to impairment from SD on the *flexibility*, *new cue*, and *new probe* indices (*F*_1,43_ ≥ 6.6, *p* ≤ 0.014), whereas subjects with the C/C genotype were particularly resilient (*F*_1,43_ ≤ 1.9, *p* ≥ 0.175). In contrast, the effect of SD on pre-switch performance relative to baseline was similar among the DRD2 genotypes in the SD group. Importantly, the effect of SD on the *vigilant attention* index was not differentially influenced by genotype, with all three genotypes (C/C, C/T, and T/T) showing vulnerability to impairment in vigilant attention (*F*_1,43_ ≥ 12.6, *p* < 0.001). In the control group, analysis of the AX-CPT-s revealed no significant DRD2 genotype effects (Fig. S2).

Further evidence that the influence of DRD2 genotype was specific to top-down attentional control was obtained from the Psychomotor Vigilance Test (PVT)^36^. The PVT, which measures vigilant attention performance, did not show resilience to SD in the C/C genotype (Fig. S3). Collectively, our results show that the preservation of AX-CPT-s post-switch performance in the C/C genotype was specific to flexibility of attentional control, and not driven by a general insensitivity to SD-induced impairment in vigilant attention. Thus, the variations in SD effects by DRD2 genotype provide additional, converging evidence of *distinct* effects of SD on attentional control.

## Discussion

Our data reveal that people under SD can maintain information in the focus of attention and anticipate likely correct responses, but their use of such a top-down attentional control strategy is less effective at preventing errors caused by competing responses. Moreover, when changing task demands require flexibility in top-down attentional control, performance under SD suffers dramatically. The finding that top-down attentional control is less effective and less flexible under SD elucidates how SD may cause perseveration and other maladaptive behaviors of sleepy people. This provides an explanation for the prevalence of critical, SD-induced errors in everyday life – from the emergency room to the boardroom – that have been difficult to explain on the basis of deficits in vigilant attention.

Our results challenge the prevailing paradigm that most if not all of the effects of sleep deprivation on cognitive performance are downstream effects of the impact of sleep deprivation on vigilant attention^2^. Our finding that subjects homozygous for the C allele of the DRD2 C957T polymorphism are resilient to SD effects on cognitive flexibility, but not vigilant attention, confirms that the effects of SD on attentional control are dissociable and not merely a downstream effect of degraded vigilant attention. This finding also sheds light on previous data showing that individual differences in the effects of SD on cognition are trait-like *but task-specific*^37^. It suggests that distinct neuronal pathways are involved in the effects of SD on cognitive flexibility^18^, specifically the striatum where the DRD2 polymorphism affects dopamine receptor D2 binding potential^33^. More generally, our data are consistent with other recent reports^14,38^ illustrating that relationships between cognition and genetic polymorphisms, which tend to require large sample sizes to detect, may emerge robustly under conditions that strongly challenge cognitive processing abilities, such as SD.

## Methods

### Study participants

The 49 volunteers who participated in the study were carefully screened. Physical exam, history, questionnaires, blood chemistry, urine drug screen, and breathalyzer test showed them to be healthy, not pregnant, and free of drugs. Polysomnography during the first night in the laboratory revealed no sleep disorders. Subjects had no history of learning disability; reported good habitual sleep of between 6 and 10 hours daily; and regularly woke up between 06:00 and 09:00. They reported no shift work within 3 months and no travel across time zones within 1 month of entering the study. They had normal or corrected-to-normal vision. Subjects maintained their regular sleep schedule, with no daytime naps, during the 7 days prior to admission into the laboratory. They refrained from alcohol, drugs (including tobacco) and caffeine during the 7 days before and during the laboratory study.

The study was approved by the Institutional Review Board (IRB) of Washington State University (WSU), and all subjects gave written informed consent. All study procedures conformed to those in the protocol approved by the WSU IRB.

### Sleep deprivation protocol

The experiment was conducted under controlled laboratory conditions, with stable ambient temperature (21 ± 1 °C). Light levels were fixed (<100 lux) during scheduled wakefulness and lights were off during scheduled sleep. Subjects were monitored continuously throughout the experiment, and no visitors, phone calls, live radio or television, or internet access were allowed. Subjects were in the laboratory for 72 hours (4 days, 3 nights). They were randomized to a SD group or a control group (approximately 2:1). Subjects in the SD group had a 10-hour baseline sleep opportunity, were subsequently kept awake for 38 hours, and then had a 10-hour recovery sleep opportunity. Subjects in the control group had a 10-hour sleep opportunity each night. All sleep opportunities were from 22:00 until 08:00.

### AX-CPT-s performance testing

The AX-CPT-s, modeled after the AX-CPT^26,27^, assessed flexible attentional control by measuring subjects’ ability to accurately identify valid and invalid cue–probe letter pairings. Letter pairs were presented with a 3-second delay between the first and second letters. Subjects were asked to respond by clicking the left and right mouse buttons to indicate valid and invalid cue–probe pairs, respectively. For example, if the letter “A” (valid cue) was immediately followed by the letter “X” (valid probe), subjects were to respond by clicking the left mouse button. For all other letter pairs (e.g., A–Y, B–X, B–Y, C–D), they were to respond by clicking the right mouse button. Subjects had 2 seconds to respond and were instructed to be as fast and accurate as possible (Fig. 1).

At the beginning of the task, subjects were told which cue–probe pair was the valid target. During each test session, subjects performed 14 practice trials, followed by 4 test blocks of 40 letter pairings each. Most of the pairings (70%) were valid. After the 4 test blocks, subjects were informed that the valid cue–probe pair was switching. For example, the letter “B” became the new cue, and the letter “Y” became the new valid probe. Some of the invalid cue–probe pairs included the presentation of the formerly valid cue and/or probe. After the switch, subjects performed 2 test blocks of 48 letter pairings each. Each of the cue–probe pairings were presented equally after the switch (Fig. 1).

Two different but equivalent versions of the AX-CPT-s were used, with the second version using “C” and “Z” as the initial valid cue and probe and “S” and “G” as the valid cue and probe after the switch. The order in which the two versions were administered was randomized. Performance on the AX-CPT-s was quantified based on signal detection theory^14^. Discriminability indices d’ were calculated for each trial block based on hit and false alarm rates for the different combinations of valid and invalid—and, after the switch, previously valid but no longer valid—cues and probes (Table 1).

The AX-CPT-s was administered at 15:30 during baseline (session 1) and again 24 hours later (session 2) while well-rested (control group) or after 31.5 hours of continuous wakefulness (SD group).

### Other cognitive performance testing

The Attention Network Test (ANT)^30^ required subjects to correctly indicate the direction that a target stimulus is pointed under three cue conditions (no cue, alerting cue, or orienting cue) and two flanker conditions (congruent or incongruent). This task provides measures of distinct aspects of attention: alerting, orienting, and management of response conflict. We used the 10-minute version of the task^31^. Each test trial began with a fixation period (+), followed by either no cue or a cue (*) presented for 100 ms and positioned in the center to alert or positioned above or below the fixation point to alert and orient to where the target stimulus was going to appear. This was followed by presentation of a line of 5 left- or right-pointing arrows, centered horizontally on the screen and located either above or below the central fixation point. Subjects were asked to focus only on the direction of the center arrow, which was the target stimulus. The surrounding arrows, which served as flankers, pointed either congruently or incongruently with respect to the target stimulus. The arrows stayed on the screen for 2 seconds. Subjects were to respond by clicking the left or right mouse button corresponding to the direction of the target stimulus (center arrow). They were instructed to be as fast and accurate as possible. During each test session, subjects performed 12 practice trials, followed by 5 test blocks of 48 trials each. No-cue and center-cue trials each occurred one-third of the time; orienting-cue trials occurred one-third of the time, with above and below the central fixation point occurring equally often. Congruent and incongruent flankers occurred equally often.

Standard performance measures^31^ were derived for the ANT. An *alerting* score was calculated by subtracting mean response time (RT) for the center cue trials from mean RT for the no-cue trials. An *orienting* score was calculated by subtracting mean RT for the spatial (above or below) cue trials from mean RT for the center cue trials. A *response conflict management* score was calculated by subtracting mean RT for congruent flanker trials from mean RT for incongruent flanker trials.

The ANT was administered at 10:30 during baseline (session 1), again 24 hours later (session 2) while well-rested (control group) or after 26.5 hours of continuous wakefulness (SD group), and once more 24 hours later (session 3) after recovery sleep.

The Psychomotor Vigilance Test (PVT), a standard measure of vigilant attention^2,37^, required subjects to respond as quickly as possible, by pressing a button, to a simple visual stimulus that occurred at random intervals of 2 to 10 seconds for 10 minutes. Subjects were instructed to be as fast as possible without making false starts. They received feedback on their response time for 1 second after each response. For the PVT, performance was quantified by the number of lapses in vigilant attention, defined as RT ≥ 500 ms.

The PVT was practiced twice on the day of admission into the laboratory, and was then administered at 09:00, 13:00, 17:00 and 21:00 during the baseline day and at the same times of day 24 hours later while well-rested (control group) or sleep-deprived (SD group).

### Genotyping

Blood samples were collected from subjects in Vacutainer tubes (Becton Dickinson, Franklin Lakes, NJ) coated with K_2_EDTA to prevent clotting. Samples were immediately aliquoted and stored at −80 °C until DNA extraction. Genomic DNA was extracted from 100 µl of red cell-depleted whole blood. Samples were assayed for the dopamine receptor D2 (DRD2) gene, which codes for the D2 receptor subtype. The DRD2 gene contains a single SNP involving a cytosine (C) to thymine (T) substitution at position 957 (C957T; chromosome 11). This polymorphism does not cause a change in the amino acid sequence. Homozygosity for the C allele (C/C) is associated with decreased D2 receptor availability and decreased dopamine binding potential in the striatum.

DRD2 C957T genotypes were assayed using the Taqman SNP Genotyping Assay (ThermoFisher Scientific, Waltham, MA) per manufacturer protocol. Samples were assayed in duplicate, with inclusion of a no-DNA negative control. Following assay completion, allelic discrimination software (MJ Opticon Monitor Analysis v3.1; Bio-Rad Laboratories, Hercules, CA) was used to identify the genotypes for each subject. The DRD2 C957T genotype distributions were as follows: C/C: 8 (7 in the SD group); T/C: 24 (15 in the SD group); T/T: 17 (12 in the SD group). The overall subject sample was found to be in Hardy-Weinberg equilibrium (*χ*^2^_1_ = 0.08, *p* = 0.79). The sample for the SD group was in Hardy-Weinberg equilibrium (*χ*^2^_1_ = 0.34, *p* = 0.57), and the sample for the control group was also in Hardy-Weinberg equilibrium (*χ*^2^_1_ = 1.28, *p* = 0.26). Allele frequencies were consistent with those reported in the literature^39,40^.

### Statistical analyses

Statistical testing of differences in task performance across AX-CPT-s d’ indices, test sessions and groups was performed using multivariate analysis of variance (MANOVA) with fixed effects for d’ index, group, and session, and their two-way and three-way interaction. Here d’ index and session were implemented as repeated measures. Statistical testing of differences in task performance across test sessions and between groups for specific AX-CPT-s d’ indices, specific ANT outcome measures, and PVT performance was performed using mixed-effects analysis of variance (ANOVA), with fixed effects for group and session and their interaction. For the PVT, time of day (09:00, 13:00, 17:00, 21:00) and its interactions with group and session were also included. Here session time of days were repeated measures. A random effect over subjects was placed on the intercept^41^. Gene analyses were performed by adding genotype as a covariate fixed effect alone and in interaction with the other effects. Head-to-head comparisons between sessions were based on *a priori* planned contrasts.

### Data sharing

Data produced in these studies will be maintained in a secure cloud-based backup system and are available on request from any legitimate academic, scientific or governmental entity. Requests should be directed to the PI at the Sleep and Performance Research Center, Washington State University, Spokane, WA.

## Electronic supplementary material

Supplementary information
