# Author Correction: Sleep deprivation diminishes attentional control effectiveness and impairs flexible adaptation to changing conditions

**DOI:** 10.1038/s41598-023-41215-0

**Published:** 2023-08-29

**Authors:** Paul Whitney, John M. Hinson, Brieann C. Satterfield, Devon A. Grant, Kimberly A. Honn, Hans P. A. Van Dongen

**Affiliations:** 1 Department of Psychology, Washington State University, Pullman, WA 99164-4820 USA; 2 Sleep and Performance Research Center and Elson S. Floyd College of Medicine, Washington State University, Spokane, WA 99210-1495 USA; 3 Department of Psychiatry, College of Medicine, University of Arizona, Tucson, AZ 85721 USA

Correction to: *Scientific Reports* 10.1038/s41598-017-16165-z, published online 22 November 2017

This Article contains an error in the Results section.

“In addition, performance decreased on *any* trial that included either an old cue, reflected in the *new cue d’ index* (*F*_1,47_ = 4.5, *p* = 0.039), or an old target probe, reflected in the *new probe d’ index* (*F*_1,47_ = 31.8, *p* < 0.001).”

should read:

“In addition, performance decreased on *any* trial that included either an old target probe, reflected in the *new cue d’ index* (*F*_1,47_ = 4.5, *p* = 0.039), or an old cue, reflected in the *new probe d’ index* (*F*_1,47_ = 31.8, *p* < 0.001).”

