# Supplementary material for: Sleep Deprivation Diminishes Attentional Control Effectiveness and Impairs Flexible Adaptation to Changing Conditions
Source: Sci Rep. 2017 Nov 22;7:16020. doi: 10.1038/s41598-017-16165-z (PMC5700060; doi:10.1038/s41598-017-16165-z)
Supplement: Supplementary file 1 — Supplementary information [file 41598_2017_16165_MOESM1_ESM.doc]

**Sleep Deprivation Diminishes Attentional Control Effectiveness and Impairs Flexible Adaptation to Changing Conditions**

**Authors:** Paul Whitney^1^, John M. Hinson^1,*^, Brieann C. Satterfield^23^, Devon A. Grant^2^, Kimberly A. Honn^2^, Hans P. A. Van Dongen^1,2^

**Affiliations:**

^1^ Department of Psychology, Washington State University, Pullman, WA 99164-4820, USA.

^2^ Sleep and Performance Research Center and Elson S. Floyd College of Medicine, Washington State University, Spokane, WA 99210-1495, USA.

^3^ Department of Psychiatry, College of Medicine, University of Arizona, Tucson, AZ 85721, USA

^*^Corresponding author. Email: [hinson@wsu.edu](mailto:hinson@wsu.edu)

**Supplementary information**

**Table S1.** Statistical results of mixed-effects analysis of variance (ANOVA) of AX-CPT-s performance for each of the d’ indices.

|  | |  | **Main**  **group effect** | | **Main**  **session effect** | | **Group by session interaction** | |
| --- | --- | --- | --- | --- | --- | --- | --- | --- |
| **Index** | | | ***F*_1,47_** | ***p*** | ***F*_1,47_** | ***p*** | ***F*_1,47_** | ***p*** |
| *Pre-switch* | *A-cue d’* | | 0.6 | 0.426 | 14.5 | <0.001 | 1.5 | 0.224 |
|  | *X-probe d’* | | 9.6 | 0.003 | 17.6 | <0.001 | 18.2 | <0.001 |
|  | *vigilant attention d’* | | 4.5 | 0.039 | 10.7 | 0.002 | 16.1 | <0.001 |
| *Post-switch* | *flexibility d’* | | 7.8 | 0.008 | 3.6 | 0.063 | 19.0 | <0.001 |
|  | *new cue d’* | | 2.5 | 0.121 | 0.2 | 0.635 | 3.5 | 0.068 |
|  | *new probe d’* | | 9.1 | 0.004 | 4.6 | 0.037 | 16.7 | <0.001 |

**Fig. S1. Performance on the ANT in the control and SD groups.** Panels show the alerting (**A**), orienting (**B**) and conflict (**C**) effects in session 1 (BL, baseline), session 2 (WR, well-rested) and session 3 (RC, recovery) in the control (*C*) group; and in session 1 (BL), session 2 (SD, sleep-deprived) and session 3 (RC) in the sleep deprivation (*SD*) group. Upward changes reflect performance degradation. Error bars are ± 1 SEM. Brackets indicate significant contrast comparing session 1 to 2 and session 2 to 3 (*p* < 0.05).


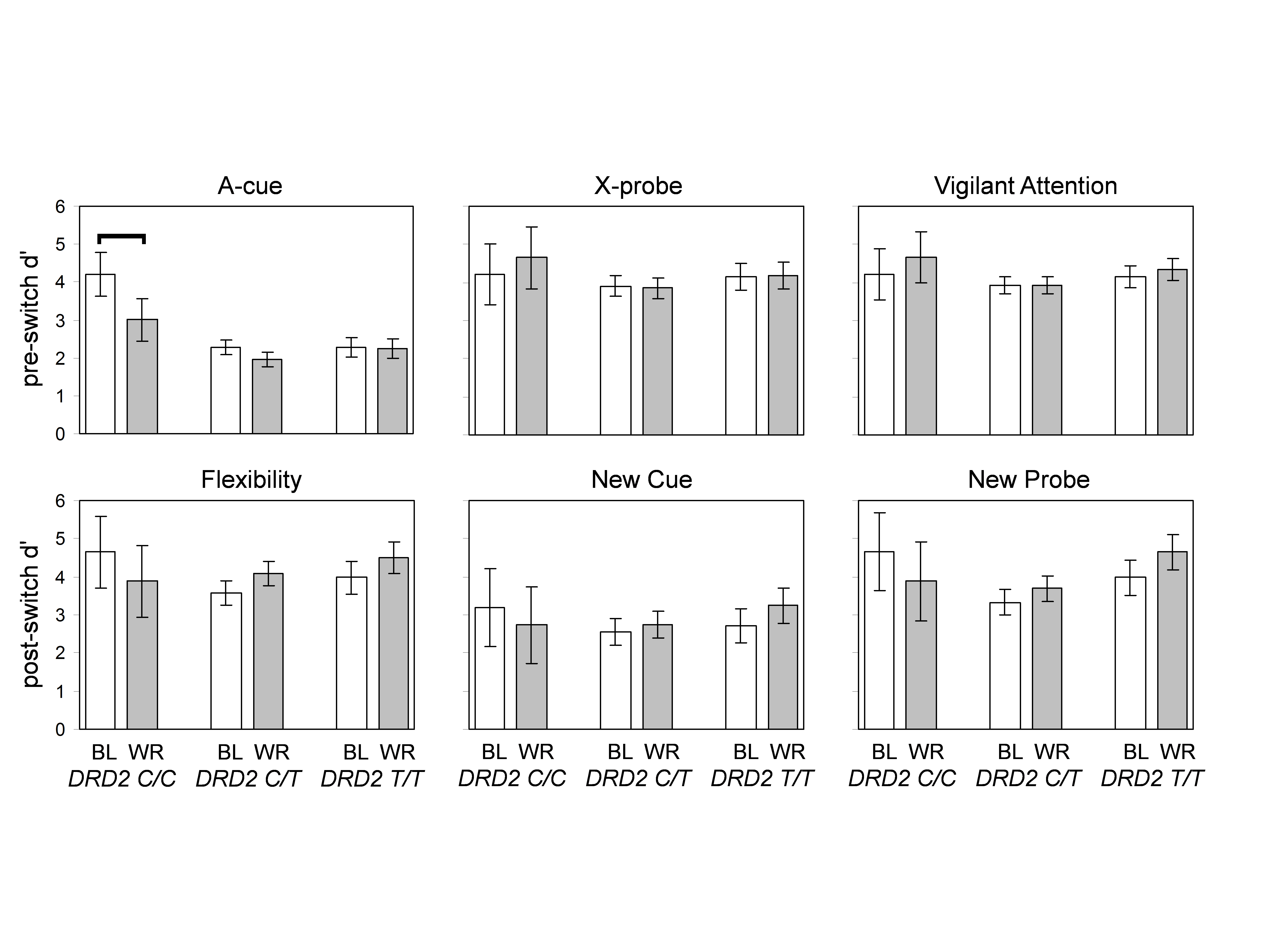


**Fig. S2. Performance on the AX-CPT-s in the control group by genotype.** Panels show d’ for each of the performance indices described in Table 1, in session 1 (BL, baseline) and session 2 (WR, well-rested) for subjects homozygous for the C allele (*C/C*), heterozygous (*C/T*), or homozygous for the T allele (*T/T*) of the DRD2 C957T polymorphism. See Table 1 for interpretation of d’ changes. Error bars are ± 1 SEM. Bracket indicates contrast comparing session 1 to 2 approaches significance (*p* = 0.098).

**Fig. S3. Performance on the PVT in the control and SD groups by genotype.** The panels show attentional lapses by genotype in the control group (**A**) and the SD group (**B**), during the baseline day (BL) and 24 hours later while well-rested (WR) or sleep-deprived (SD), for subjects homozygous for the C allele (*C/C*), heterozygous (*C/T*), or homozygous for the T allele (*T/T*) of the DRD2 C957T polymorphism. Upward changes reflect performance degradation. Error bars are ± 1 SEM. Brackets indicate statistically significant contrast comparing performance during the baseline day to 24 hours later (*p* < 0.05).
